# A combined intervention strategy to increase linkage to and retention in substance use treatment for individuals accessing hospital-based services: study protocol

**DOI:** 10.3389/fpsyt.2024.1330436

**Published:** 2024-05-16

**Authors:** A. S. Crisanti, K. Page, J. L. Saavedra, T. Kincaid, C. M. Caswell, V. A. Waldorf

**Affiliations:** ^1^Department of Psychiatry and Behavioral Sciences, University of New Mexico Health Sciences Center, Albuquerque, NM, United States; ^2^Department of Internal Medicine, University of New Mexico Health Sciences Center, Albuquerque, NM, United States

**Keywords:** peer support workers, rural behavioral health telehealth, addiction treatment, trauma-specific treatment, study protocol

## Abstract

**Background:**

In 2020, New Mexico had the highest alcohol related death and the 11th highest drug overdose rate in the U.S. Towards the long-term goal of addressing this public health problem, we are implementing and evaluating an multi-level intervention designed to identify adults at risk of substance use disorder (SUD) and encourage linkage to and retention in treatment. The first level includes equipping the ED and medical inpatient units of a safety-net hospital with a method to screen individuals at risk of a SUD. The second level includes Seeking Safety (SS), a trauma-specific treatment for PTSD and SUD; and pharmacotherapy for SUD. Motivational Interviewing (MI) is used throughout both levels. Using the SPIRIT guidelines and checklist, this study protocol describes the multi-level intervention and the methodology we are using to assess feasibility and effectiveness.

**Methods:**

We are using a Type 1 hybrid implementation design with a non-randomized approach (ISRCTN registration # ISRCTN33100750). We aim to enroll 110 adults ( ≧18 ) who screen positive for unhealthy use of alcohol, prescription medications (used nonmedically) and/or illicit drugs. Peer support workers are responsible for screening, using MI to increase engagement in screening and treatment and delivery of SS. Pharmacotherapy is provided by addiction clinical specialists. Treatment is provided post hospital discharge via telehealth to increase access to care. Participants are identified through (1) review of electronic health records for individuals with a chief or secondary complaint or mental health condition relating to alcohol and/or other drug use, (2) referrals from clinical staff and (3) screening in the ED and medical inpatient units. Feasibility is being measured through process data. Effectiveness will be determined by changes in two primary outcomes: (i) PTSD symptom severity; and (ii) substance use.

**Discussion:**

Our study will expand on research related to the implementation of treatment strategies for patients presenting at EDs and admitted to medical inpatients units wherein there is a significant window of opportunity to link patients with follow-up behavioral and clinical services for alcohol and/or drug misuse. The challenges associated with implementation and strategies that have been helpful to address these challenges will further inform the field.

## Background

In 2020, New Mexico (NM) had the highest alcohol related death rate in the U.S.; 86.6 deaths per 100,000 compared to 41.5 nationally (1). It also had the 11th highest drug overdose rate in the U.S., with 801 total deaths due to drug overdose (2). Recent data indicate an even worsening trend, with drug overdose rates increasing in NM at a far greater rate compared to the rest of the U.S. The drug overdose death rate in NM increased from 26.3 per 100,000 in 2011 to 51.6 per 100,000 in 2021. In contrast, the drug overdose death rate in the U.S. was 13.2 per 100,000 in 2011 and 32.4 per 100,000 in 2021 (2). Noteworthy, is that “approximately 75% of drug overdose deaths in 2021 involved at least one opioid and 66% of deaths involved synthetic opioids (e.g., illicitly manufactured fentanyls)” (3)Among the U.S., NM has the third highest percentage of Native Americans (12%) (4) and consistent with national level data, Native Americans in NM have a much higher risk of alcohol poisoning deaths than any other race group (5).

The consequences of excessive substance use have been far reaching for New Mexicans, including high rates of domestic violence, fatal crashes involving intoxicated drivers and deaths due to alcoholic liver disease, cirrhosis, unintentional injuries and suicide (6). Our ability to improve this public health problem is based on the availability of evidence-based care, as well as the success to which those with substance use disorders (SUD) are identified clinically, are referred to treatment, can access treatment and initiate treatment.

Individuals with a SUD often present to the emergency department (ED) or are admitted to an inpatient unit of a hospital for a variety of reasons, including substance use related injuries, infections and overdoses (7). Nearly half of all ED visits in the US are categorized as being related to SUDs (8) and substance abuse related admissions to intensive care or other hospital medical units are widespread (9). As a result, hospital-based settings provide an opportunity to actively engage patients in a discussion about the need for treatment and link them into appropriate services and support (8).

Unfortunately, many EDs and inpatient units struggle with not having enough staff or staff with expertise or experience to effectively engage with individuals with SUDs, including telling them about their treatment options and linking them to care in a non-rushed and non-judgmental way in times of crisis (10–15). To address this need, programs have been placing peer support workers (PSWs) within EDs to support people with SUD (16). PSWs are “people who have been successful in the recovery process who help others experiencing similar situations” (17). Other terms for this workforce include peer recovery support services, peer recovery coach/specialist and peer navigator/advocate.

Provision of peer support services is an evidence-based practice for SUD care and evidence for the benefits of these services is accruing (13, 18–21). When placed in the ED, PSWs have been associated with greater SUD treatment engagement, especially with high-risk populations, and shorter days to initiation for substance use treatment (13, 15, 18, 22–24). However, there is scant data on the feasibility of employing PSWs in the ED and medical inpatient units who can support screening and linkage to care provided via telehealth. Furthermore, the studies conducted thus far have focused on PSW interventions that have been implemented in EDs that serve mostly urban populations. Research on the effectiveness of these interventions within hospitals that serve rural and tribal populations is needed, given the high rates of SUD in these communities and the increasing use of EDs among individuals with SUD, especially from rural areas (25).

To the extent possible, an unencumbered transition of care from the hospital to outpatient treatment is critical for individuals with SUD who want to engage in treatment for their SUD before being discharged. This includes making access to treatment as easy as possible, especially for groups more likely to face barriers to accessing care. Telehealth has been shown to increase access to addiction treatment, particularly in rural areas where services within communities are limited and transportation to services outside of the community is unavailable or difficult (26, 27). Telehealth for SUD treatment has also been shown to be associated with increased retention in SUD treatment and increased therapeutic alliance (28).

To increase the likelihood that (1) those at risk of a SUD who present to the ED or are admitted to an inpatient unit are identified and referred to treatment, and (2) those determined eligible are referred to and can access treatment, we are conducting a multi-level implementation study assessing the feasibility and effectiveness of a combination intervention strategy comprised of several evidence-based and practical interventions (29).

The objective of our study is to evaluate the feasibility and effectiveness of an integratedmulti-level intervention that identifies individuals at risk of a SUD and encourages linkage to and retention in community-based SUD treatment. Using the SPIRIT guidelines and checklist (http://www.spirit-statement.org/), this study protocol describes the multi-level intervention and the methodology we are using to assess feasibility and effectiveness.

## Methods: participants, multi-level intervention, and outcomes

### Study design

**Figure 1:**
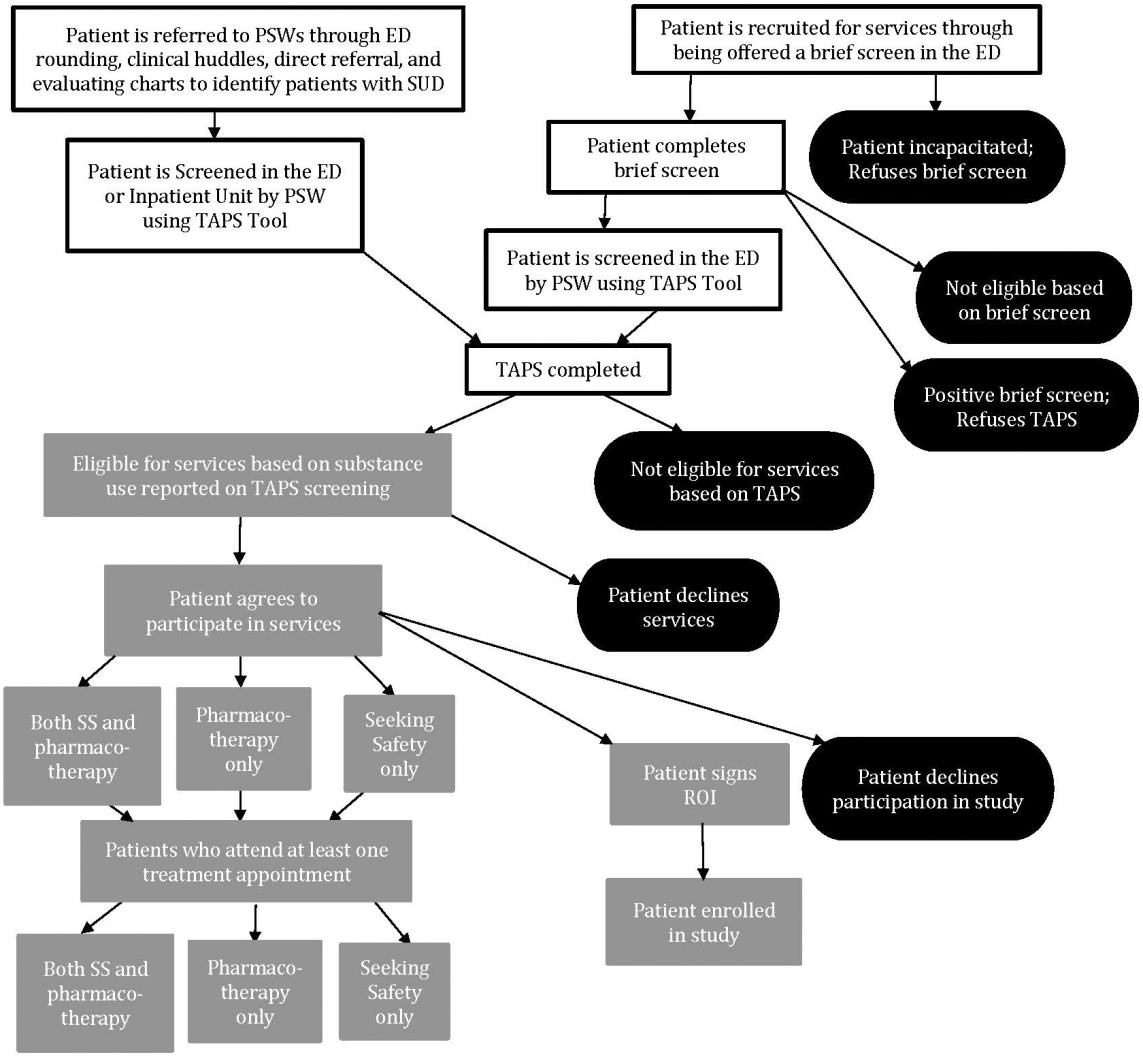
Overview of Participant Flow Through the Multi-Level Intervention and Study Enrollment*. *Footnote: The first level of the multi-level intervention includes equipping the ED and medical inpatient units of a safety-net hospital with a method to screen individuals at risk of a SUD. The second level includes Seeking Safety (SS), a trauma-specific treatment for PTSD and SUD; and pharmacotherapy for SUD. Motivational Interviewing (MI) is used throughout both levels.

This study uses a Type 1 hybrid implementation design with a non-randomized approach (30) (see **Figure 1**). While the primary focus of our study is to test the intervention, we are also very interested in understanding implementation related factors. As a result, we are collecting process data along every step of **Figure 1**, including for example, the number of referrals and patients screened, number of patients determined eligible and reasons for ineligibility, number of patients who agree to treatment and attend at least one appointment.

### Study site

The study site is a safety-net hospital located in Sandoval County, NM. Sandoval County is the 4^th^ most populous county in NM, with 131,561 residents and covering 3,716 square miles. 40% of the residents are Hispanic and 14.1% are Native American (NA). Most health care services in Sandoval County are available in the “metro” corner, which is only 150 square miles and where two-thirds of the county’s population reside. The “nonmetro” area is home to 13 sovereign tribal nations, including 10 Pueblos, two Navajo chapter houses and a portion of the Jicarilla Apache reservation. This is the second highest number of reservations of any county in the U.S. The nonmetro area covers over 3,500 square miles and residents struggle with limited access to healthcare and high rates of poverty, mental health problems, substance use and suicide. In Sandoval County, the alcohol related death rate for NA is 174.2 per 100,000 compared to 56.0 and 35.1 per 100,000 for the overall state and U.S., respectively (31). Sandoval County is one of the top five counties with the highest number of methamphetamine-involved overdose deaths and has the 2nd highest number of opioid overdose related ED visits in the state (N=363, 6.1%) (31). The study period is October 2022 – September 2024. This study has been registered on the ISRCTN registry # ISRCTN33100750.

The study site hospital houses 72 inpatient beds, divided between two medical surgical units and one intensive care unit. The ED has 20 licensed beds. Fiscal year 2021 data from the study site hospital showed that 1,366 patients, representing 1,958 visits, accessed the ED with a SUD diagnosis (including alcohol use disorder). 75% of all inpatient discharges from the medical units with a behavioral health diagnosis had a SUD. Compared to 2020, the number of individuals with a primary diagnosis of SUD increased by 75% in the ED and 48% in the medical inpatient units in 2021. For the past three years, Native Americans comprised 56% of all admissions to the ED for alcohol use disorder. Furthermore, 30% of the ED behavioral health visits in 2021 were from designated rural communities of Sandoval County.

### Eligibility criteria

Participants are adults 18 years of age or older who present to the ED or are admitted to a medical inpatient unit and who have screened positive for unhealthy use of alcohol, prescription medications (used nonmedically) and/or illicit drugs on the National Institute on Drug Abuse Tobacco, Alcohol, Prescription medication and other Substance Use (TAPS) (32). Individuals with unhealthy tobacco use only are excluded from the target population. Participants are excluded from the program if they are under 18 years of age, cannot speak English, are cognitively-impaired and therefore unable to provide consent, or do not wish to participate in treatment. Based on previous research with this population, the percent of non-English speaking individuals in our target population is less than one percent. Individuals who are not included in the study are still eligible to receive services through SRMC TH-SUD treatment and any other services at SRMC. The PSWs are responsible for conducting the screening and determining eligibility.

### Multi-level intervention

Our multi-level intervention combines several evidence-based and practical interventions (see **Figure 1**). The first level includes equipping the ED and medical inpatient units of a safety-net hospital with a method to screen individuals at risk of a SUD. The second level includes Seeking Safety (SS), a trauma-specific treatment for PTSD and SUD; and pharmacotherapy for SUD. Motivational Interviewing (MI) is used throughout both levels. Each of these interventions will be described in more detail below. SS and pharmacotherapy for SUD is provided post discharge via telehealth to increase access to care.

### Hiring and training PSWs

PSWs are key to the implementation of our intervention. PSWs are hired through the study site hospital human resource office. Job postings were developed specifically for PSWs positions for this study. Both full-time and part-time employment opportunities are available for PSWs. Applicants that reflect the race/ethnicity of the target population are given priority as well as individuals in recovery from substance use disorders. All PSWs receive initial and ongoing training for both MI and SS. The MI training consists of two half-days; Part One covers the SPIRIT of Motivational Interviewing, the importance of accurate empathy, understanding ambivalence and resistance, and basic skills: OARS (open ended questions, affirmations, reflections, summary). Part Two reviews SPIRIT, OARS and ambivalence and teaches the basic MI skill of CHANGE Talk using DARN CATS, skillful responses and double-sided reflections. The SS initial training consists of two half-days covering an introduction to the intervention as well as its implementation. Ongoing training is provided for PSWs in both MI and SS to increase fidelity to the models. Ongoing MI training is provided through monthly learning communities which cover topics such as recognizing the stages of change and simple and complex reflections. Ongoing SS training is provided through weekly practice sessions (including role playing) between the clinical supervisor and PSWs. PSWs also complete a number of other trainings to prepare them for working in a rural hospital environment and with the target population, such as Cultural Sensitivity, Clinical Documentation, Suicidal Ideation Response for Telehealth, pharmacotherapy for SUD, and TAPS administration. Finally, PSWs meet with a dedicated clinical supervisor once per week to discuss any difficulties with patients or questions about clinical processes.

### Screening for SUD in the hospital

The identification of individuals at risk of SUD in the ED and medical inpatient units is a major component of the intervention. PSWs use the TAPS screening tool to determine study eligibility. The TAPS includes two parts: firstly, five questions that ask about substance use (i.e., tobacco, alcohol, non-medical use of prescription drugs, and illicit drugs) in the past 12 months, and secondarily among those who screen positive on part 1, nine questions about substance use in the past three months (32).

There are multiple ways in which individuals are identified to complete the TAPS. The first way is with a brief screening tool that is completed on individuals 18 years of age or older who present to the ED. The brief screening tool includes two questions: (1) how many times in the past year have you had 4 or more drinks in one day, and (2) how many times in the past year have you used a recreational drug or used a prescription medication for non-medical reasons? Recreational drugs include methamphetamines (speed, crystal) cannabis (marijuana, pot), inhalants (paint thinner, aerosol, glue), tranquilizers (benzodiazepines), barbiturates, cocaine, ecstasy, hallucinogens (LSD, mushrooms) or opioids (fentanyl, heroin). Individuals whose response is >1 on the brief screen are invited to complete the TAPS. The second way PSWs identify individuals to complete the TAPS is through targeted identification of ED patients and inpatients with a chief or secondary complaint or mental health condition relating to alcohol and/or other drug use. To do this, PSWs review the electronic health records of ED patients and inpatients for whom substance use was a presenting complaint. PSWs also attend ED rounding, huddles (that occur during shift changes where the PSWs meet with the incoming ED providers) and obtain referrals from clinical staff through TigerConnect, which is a secure real-time messaging app used by hospital staff.

### Communication post screening

PSWs are responsible for informing those determined at risk of SUD about the results of the TAPS screen and about the specifics of the community-based intervention, including the treatment options, the method of delivery (telehealth) and that because the intervention is grant funded the treatment that they are being offered is free. When sharing the results of the TAPS screen and the reason for moving on from the first part of the TAPS to the second part, PSWs are required to follow a script to ensure consistency among the team. PSWs provide patients a treatment menu that briefly describes both interventions, SS and pharmacotherapy, with words and pictures. Patients are asked to choose which services they would like to participate in; SS only, pharmacotherapy only or both. PSWs also provide patients with a “discharge packet” that includes instructions on how to access telehealth, hard copies of the SS materials, and a discharge summary which lists the services they agreed to participate in as well as contact information for the PSW they met with.

Assessing Barriers to Online Treatment. Because both SS and pharmacotherapy for SUD is provided via telehealth, PSWs are responsible for checking with patients about their access to treatment online. PSWs can provide patients who do not have a way to access treatment a cell phone with monthly paid service.

### Motivational interviewing

PSWs use Motivational Interviewing (MI) to encourage patients to complete the brief screen and the TAPs. They also use MI to engage those determined eligible into treatment. MI is a brief intervention that helps individuals resolve ambivalence about behavior change by eliciting and enhancing intrinsic motivation for change (33). A large body of research supports the effectiveness of MI for substance use (34).

### Seeking safety

SS is an evidence-based, present-focused, manualized brief treatment that uses cognitive-behavioral approaches designed to target trauma, PTSD and SUDs (35). SS provides coping skills that enable participants to cope in safe ways without substances and without harmful behaviors. The literature on the effectiveness of SS is vast and includes evidence on the effective delivery of SS by PSWs (36). Studies have shown positive outcomes including decreases in substance use, PTSD and trauma related symptoms, and improvements in social adjustment, suicidal thoughts, coping, sense of meaning and quality of life (37–39). SS includes 25 topics that cover behavioral, interpersonal and cognitive domains. However, because SS is designed for a high level of flexibility and topic order can vary, similar to other studies of SS, we selected 10 modules most relevant to our target population to begin with (36). The modules include: (1) Safety, (2) PTSD: Taking Back Your Power, (3) When Substances Control You, (4) Detaching from Emotional Pain, (5) Asking for Help, (6) Coping with Triggers, (7) Discovery, (8) Setting Boundaries in Relationships, (9) Recovery Thinking, (10) Honesty. With up to 97.4% of individuals with SUD having a history of trauma, a trauma-specific focused intervention can be a critical component of SUD treatment (40). PSWs are responsible for delivering SS one-on-one with patients via telehealth. Appointments are scheduled by PSWs or a Patient Assistant Representative. SS materials are either sent to the patients electronically or mailed depending on preference.

### Pharmacotherapy for SUD

Pharmacotherapy for SUD involves the use of FDA approved medications that are often combined with counseling and behavioral therapies for people diagnosed with SUD. Pharmacotherapy for SUD has proven to be clinically effective with respect to reducing substance use, cravings, withdrawal symptoms and preventing overdoses (41, 42). Evidence also suggests that combining counseling services, including MI, with pharmacotherapy for SUD, improves treatment retention (43). FDA approved drugs for opioid use disorder include methadone, buprenorphine, and naltrexone. While pharmacotherapy for SUD is mostly known for opioid use disorder, because of the large prevalence of alcohol use disorder among the target population, our addiction specialists are also offering approved FDA medications for AUD, including acamprosate, disulfiram, and naltrexone. For short term withdrawal, prescriptions include gabapentin and carbamazepine.

### Outcomes

**Table 1** provides an overview of our primary, secondary and other outcomes, including the specific tool used to measure the outcome and measurement time point.

**Table 1:** Overview of data collection.

Domain	Data Collection Tool	Time Point	Variable Type
Post-Traumatic Stress Disorder Symptom Severity	PTSD CheckList – Civilian Version (PCL-C) (44)	Baseline and every 60 days	Primary Outcome
Substance Use	Addiction Severity Index (45)	Baseline and every 60 days	Primary Outcome
Demographics	Age, race, ethnicity, gender (assigned at birth and current identity), relationship status, level of education, income level, employment status, health insurance status and provider, housing status	Baseline evaluation	Covariates
Criminal Activity Involvement	Criminal Justice Questionnaire (developed by first and second author)	Baseline and every 60 days	Secondary Outcome and Predictor
Motivation to Change	The Change Questionnaire (46)	Baseline and every 60 days	Secondary Outcome and Predictor
Treatment Satisfaction	Client Satisfaction Questionnaire (CSQ-8) (47)	Baseline and every 60 days	Secondary Outcome and Predictor
Quality of Life	36-Item Short Form Survey Instrument (SF-36) (48)	Baseline and every 60 days	Secondary Outcome and Covariate
Past Barriers to Substance Use Treatment	Barriers Questionnaire (49)	Baseline and every 60 days	Secondary Outcome and Covariate
Perception of Recovery	The Questionnaire about the Process of Recovery (QPR) (50)	Baseline and every 60 days	Secondary Outcome and Covariate
Self-Stigma	Substance Abuse Self-Stigma Scale (51)	Baseline and every 60 days	Secondary Outcome and Covariate
Substance Craving	3-Item Substance Craving Scale (52)	Baseline and every 60 days	Secondary Outcome and Covariate
Emergency Department Utilization	Services Use Questionnaire and EHR (developed by first and second author)	Baseline and every 60 days	Secondary Outcome and Covariate
Hospital and/or treatment facility utilization	Services Use Questionnaire and EHR (developed by first and second author)	Baseline and every 60 days	Secondary Outcome and Covariate

### Sample size

We estimated our sample size using GPower (version 3.1.9.7) and focused on a meaningful change from baseline to six-months on the primary outcome PTSD symptom severity via a t-test means difference between two dependent means. The expected mean change in PTSD symptom severity as measured by the PCL-C was based on findings from research conducted by the first author in which PSWs implemented SS to a similar population (36). Assuming a moderate effect size, with an alpha = 0.05, and power = 0.80 the GPower analysis determined that a total sample size of 101 would be needed.

## Methods: data collection, management and analysis

Data collection consists of activity logs completed by project personnel, surveys completed by hospital personnel, participant interviews, and extraction of patient-level data from medical records.

### Activity logs

To determine the feasibility of our intervention, process data are being collected by project personnel via activity logs on the number of (1) brief screenings, (2) TAPS, (3) referrals from hospital staff, (4) individuals determined eligible for SUD care, (5) individuals who engage in treatment and type of treatment (i.e., SS and/or pharmacotherapy), and (5) the number of SS and/or pharmacotherapy sessions completed.

### Surveys completed by hospital staff and PSWs

To further our understanding of feasibility, we are also collecting data from hospital personnel on perceived acceptability, appropriateness, and feasibility of the screening component of our intervention. Acceptability refers to how those targeted and involved in implementing the program react to the screening. Appropriateness refers to the extent to which the screening fits within the ED and medical inpatient settings. Feasibility refers to the extent to which the screening can be successfully implemented within the ED and medical inpatient settings. These constructs will be measured by three established self-report measures: the (i) Acceptability of Intervention Measure (AIM), (ii) Intervention Appropriateness Measure (IAM), and (iii) Feasibility of Intervention Measure (FIM). The AIM, IAM and FIM include 4 items rated on a 5-point Likert scale ranging from “completely disagree” to “completely agree” (53). Higher scores indicate higher perceptions of acceptability, appropriateness, and feasibility. The measures take approximately one minute each to complete. They will be completed online by PSWs on the study team and all providers within the ED and inpatient settings, including nurses, emergency physicians, inpatient physicians and other clinical support staff. Since these instruments were not designed to be completed by those who receive the intervention, we intentionally included the Client Satisfaction Questionnaire-8 [75} as part of the battery of measures (see **Table 1**) to provide an opportunity for participants enrolled in the study to voice their opinions about the acceptability of the intervention.

### Participant interviews and consent

Once an individual agrees to engage in treatment, the PSW provides details about the opportunity to participate in a prospective follow-up cohort study. Individuals who are interested in participating sign a release of information agreeing to be contacted by a member of the research team. A member of the research team is then responsible for contacting individuals to provide further information about the study, obtaining consent for those who agree to participate and conducting baseline and follow-up interviews. Follow-up interviews are being conducted every 60-days post baseline. The consent process and all data collection is conducted by phone or a HIPAA compliant video conferencing platform. Individuals are compensated for their participation in the study with a $60 merchandise gift card for completing the baseline interview and $90 for every follow up assessment. The research team makes multiple attempts at varied times of the day and day of the week to consent individuals and complete baseline and follow up interviews. Participants are contacted via phone, text, mail and email. Participants are also offered a phone with monthly cell phone service to increase the likelihood of their participation in the study. Interviews range between 45 and 60 minutes. **Table 1** provides an overview of all data being collected during participant interviews, including the data collection tools and time point.

### Data management

Participants are assigned a participant ID number at the first stage of data processing. The participant ID number is then added to an excel file which links the participant’s identifying information to their corresponding ID number. The excel file is kept on password protected computers and the linking information will be deleted at the end of data collection. Data are never directly linked to participant identifying data. Only research team members have access to the participant ID numbers. All data are entered into a REDCap database. The REDCap database is also password protected and only the research team has access to the data. All data will be maintained and archived or destroyed for six years after the study closes then will be destroyed.

### Statistical methods

#### Feasibility

To evaluate the feasibility of our intervention, we will create a scale value for each the AIM, IAM and FIM by averaging responses. We will also summarize the number and corresponding percentages of (i) brief screens and TAPS completed, (ii) individuals determined eligible, (ii) individuals determined ineligible and reasons for ineligibility, (iii) individuals who agree to participate in treatment, (iv) types of services individuals agree to (SS and or pharmacotherapy for SUD), (iv) types of services individuals engage in, (v) SS and pharmacotherapy sessions completed. For all of these data points, we will also stratify data by gender, ethnicity, race and primary substance of use.

#### Effectiveness of the multi-level intervention

Two primary outcomes will inform our assessment of effectiveness: change in PTSD symptom severity [as measured by the Post-Traumatic Symptom Checklist (44)] and change in substance use [as measured by the Addiction Severity Index (45)]. We will use a pre-post intervention multilevel regression model analysis to examine the change in our primary outcomes of interest. The goal of the multilevel models will be to look for significant and meaningful change in the pre-post scores of our primary outcomes, PTSD symptom severity and substance use, based on the impact of the client’s predictor variables; criminal justice involvement, motivation to change and client treatment satisfaction (see **Table 1**).

To assess the impact of our covariates and secondary outcomes of interest, we will apply a multi-step approach to examine the significance of our independent variables. First, we will test for significant pre-post differences in our secondary outcomes to assess the impact of the variables from the start to finish of the client’s time in the program using parametric paired t-tests. Results of the paired tests will provide results to the significance of the impact pre-post on the client’s perceptions of the program and will provide validation of using the markers as covariates in the regression model. Second, we will apply two change-score multilevel regression models; one for the PTSD symptom severity outcome and one for the substance use outcome, with control variables (age, race, ethnicity, gender, relationship status, level of education, income level, employment status, health insurance status, provider and housing status), significant secondary outcome variables (quality of life, past barriers to substance use treatment, perception of recovery, self-stigma, substance craving, emergency department utilization and hospital treatment facility utilization) and predictor variables mentioned above. Examination of the multilevel regression model will allow for examination of client level deviation of the outcome variables by the time and predictor variables. *Post-hoc* analysis of the correlation matrix and pairwise t-tests of secondary outcomes and pre-post measurement will be examined to understand the impact of the markers in relation to the full scale of the data. Models will be used to assess for statistical robustness using the variance inflation factor for multicollinearity and appropriate error distribution tables. Latent-trait variables will be assessed for internal validity using exploratory factor analysis and reliability using Cronbach’s Alpha measure of internal consistency. While the primary outcome endpoint will be 120 days post-baseline, we will conduct secondary analyses to examine outcomes for participants using the last follow-up date post-baseline.

### Data monitoring

#### Data monitoring

A data monitoring committee was deemed unnecessary from the Institutional Review Board of the University of New Mexico Human Research Review Committee in that the study does not involve more than minimal risk to subjects.

#### Harms

If an adverse event is reported, the evaluation coordinator consults with the PI to address the issue, and the PI will report the event to the Institutional Review Board of the University of New Mexico Human Research Review Committee.

#### Auditing

The Clinical Supervisor audits the clinical records in the EHR on a weekly basis to ensure the PSWs are adhering to established clinical documentation practices. All paper and digital research records are reviewed for accuracy on a quarterly basis and discrepancies are noted, discussed and corrected.

### Ethics and dissemination

#### Research ethics approval

The study protocol, including recruitment, consent and data collection tools was reviewed and approved by the Institutional Review Board of the University of New Mexico Human Research Review Committee (HRRC 19–174). All participants provide informed written consent prior to enrollment in the study.

#### Protocol amendments

All protocol amendments are submitted to and approved by the University of New Mexico Human Research Review Committee.

#### Confidentiality

To assure confidentiality all study personnel are trained and certified in basic human subject’s research protections by the University of New Mexico Human Research Review Committee CITI Training Program. In addition, the PI administers ongoing training and supervision of the evaluation coordinator to assure confidentiality and privacy for participants and participant data.

#### Data sharing

The datasets generated during and/or analyzed during the current study are not expected to be made available due to not obtaining consent from participants during the consent process to share raw data beyond the purposes of the main study.

## Discussion

Our study will expand on previous research related to the implementation of treatment strategies for patients presenting at EDs and admitted to medical inpatients units wherein there is a significant window of opportunity to link patients with follow-up behavioral and clinical services for alcohol and/or drug misuse. Study findings have the potential to inform hospital-based approaches to the identification of individuals at risk of a SUD and their linkage to and retention in SUD treatment. Each intervention component in our multi-level intervention strategy has proven to be effective for our target population but information is lacking on whether all these approaches together are feasible and effective in improving patient health outcomes. Combined intervention strategies have been successful in HIV research (29, 54, 55) but have not been widely implemented or evaluated in the area of addiction (56).

Three main challenges have surfaced early on in the implementation of this study that have resulted in lower than anticipated enrollment of individuals in treatment and ultimately in the study. The first challenge relates to the high rate of PSW turnover. Over a period of six months, we have hired seven PSWs, of which four have resigned and two have been terminated from hospital employment. Reasons cited for resignation are consistent with those identified in the literature about challenges integrating PSWs in hospitals and other health care settings (e.g., outpatient, primary care) and include lack of integration, stigma, triggers impacting recovery, shifts that extend beyond regular work hours (e.g., 9–5), low salary coupled with high job demands and a challenging work environment and patient population (57, 58). A review of implementation challenges and recommendations for employing PSWs in EDs to support patients presenting after an opioid-related overdose (59), emphasized the importance of embedding PSWs in the work structure and setting. In practice, this would include having a designated workspace for PSWs in the ED and inpatient unit and delineating a detailed workflow. While identifying a free space for non-clinical use within these settings is often challenging, it is important to do so in that clinicians working in EDs and with PSWs have reported that integration of PSWs into the system of care is much easier when they are visible and available (60). With respect to stigma, some of our PSWs report being impacted by the stigma that they observed by staff in the ED towards those they were assisting. This stigma creates much distress and, in some cases, has served as a trigger and a threat to our PSW’s recovery in that it has brought back traumatic memories of how they, themselves, were treated by ED staff.

Several studies have reported low pay rates as a barrier to hiring and retaining PSWs (61–64). EDs are one of the most high-pressure environments within any hospital; the busy and constantly changing environment can be stressful for many people to work in, including PSWs. We have implemented several strategies to address these challenges. First, we identified a designated spot for our PSWs to work from and documented a detailed workflow. To increase the likelihood of integration of our PSWs within the ED and inpatient units, we have identified a designated workspace in the ED and also provide scrubs for our PSWs to wear, should they choose to do so. To address stigma, we have conducted widespread education among hospital personnel focusing on the role of PSWs, the benefits of including PSWs on a treatment team, risk factors associated with SUD and rates of recovery from a SUD when provided access to evidence-based treatment. Based on a review of peak hours in our ED, PSWs provide coverage seven days a week from 7:00 am to 6:30 pm. To increase the likelihood of success of our PSWs, we offer full-time as well as part-time positions and hire those who have a longer history of recovery. We also provide extensive initial and ongoing training; something that has been identified to be important to PSWs (65, 66). During the interview process, we focus on openness with applicants, including a transparent discussion around the challenges of working in the ED (e.g., ED pace and unpredictability, need for flexibility and task-switching) and with this patient population.

The second challenge is stigma towards individuals with SUD among providers in the ED, including emergency physicians and nursing staff. Stigma toward people who use substances (PWUS), including alcohol and illicit drugs arises from multiple sources, including policies and individuals who carry out policies (“structural stigma”) and health professionals (“provider-based stigma”). These types of stigmas can intersect with each other and compound negative effects (67). When PWUS anticipate or experience stigma, it impedes access to care, including pharmacotherapy for SUD (68), health services (69–72) and harm reduction programs (73). We have implemented two targeted strategies to address stigma among our hospital-based providers. The first strategy is developing laminated posters (48” x 60”) of success stories of several patients who have been enrolled into our study thus far and hanging them on walls in the ED and on an inpatient unit. The posters provide an overview of their history of addiction and how and why they ended up at the hospital. The poster also highlights improvements from baseline to follow-up (60 days and 120 days) in PTSD symptom severity, and drug and/or alcohol use and other key outcomes. Noteworthy is that the poster includes direct quotes from the patients that we are showcasing about the direct impact that the PSW has had on their lives and the importance of health care providers in supporting their recovery. The second strategy that we have implemented is a “Change the World” initiative that publicly recognizes ED and inpatient unit staff who (1) are committed to the highest quality care for individuals with SUD, and (2) have been noticed first-hand by our PSWs as treating individuals with SUD with kindness and respect. On a weekly basis, the team identifies hospital staff who fit either of these criteria and then one of our PSWs provides them with a personalized laminated globe that is signed by all of our clinical team and says “How Do You Change the World: One Person at a Time”. Since implementing the campaign, we have seen laminated Change the World Globes hanging on walls in physician and nursing staff cubicles and used as bookmarks.

The third challenge is the difficulty in engaging and retaining individuals in treatment. Challenges to substance use treatment engagement and retention are common (74, 75). In this particular study, while the participants have presented to the ED for injuries or illness related to the SUD (e.g., cirrhosis, pancreatitis), they may or may not make this connection and therefore may not be considering treatment for SUD. This could have a subsequent impact on their motivation for change, especially during the contacts that occur after their hospital encounter. It is also possible that within the initial meeting the PSWs do not have the opportunity to establish adequate rapport to assure that the relationship is of enough importance to ensure participant’s continued investment in further interactions. Examples of variables that may have impacted this are length of time the individual had been in the ED or inpatient unit, and the patient’s lack of alertness - due to intoxication and/or medications - during conversations with the PSW. This may account for some participants reportedly not remembering who the PSWs are when they are contacted post discharge and showing a reluctance to engage. Research on barriers to substance use treatment is extensive, with a recent overview of systematic reviews reporting 37 structural barriers (e.g., long waiting lists, expensive costs, insufficient options for treatment), 21 individual barriers (e.g., fear of stigma, motivational factors, psychiatric comorbidities), and 19 social barriers (e.g., difficulties with establishing a non-drug use network of friends, no supportive family) (76). There are barriers specific to our target population that are noteworthy. First, given that individuals are being recruited from the ED or medical inpatient, once discharged they are likely dealing with complex health and life circumstances that take priority over SUD treatment (77). Second, our intervention is based in a hospital that serves rural and tribal communities, and in both of these communities stigma, misinformation, mistrust and the lack of anonymity discourages individuals from seeking care (78, 79). Third, while providing services via telehealth could be perceived as being within the community, it may be that for certain participants, their preference may still be community-based providers and face-to-face interactions, versus this model of care. Finally, some of our patients from rural areas have had difficulty connecting to telehealth services because of not having broadband internet service; a challenge that has been reported in the literature on barriers to telemedicine in tribal communities (80). We have implemented several strategies to reduce barriers to care and hopefully decrease the low engagement and retention in treatment rates. First, when needed, we are providing individuals with cell phones and paid monthly cell phone plans so that they can access care provided via telehealth and to stay in contact with their PSW as well as research team. The phone comes preloaded with contact numbers for their PSW, the 988-crisis line, and Zoom and Teladoc apps to access telehealth appointments. The client has unlimited talk, text and data as long as they are engaging in services. If a client stops responding to calls and messages, they are given multiple reminders to re-engage and after six weeks of non-responsiveness, phone service is discontinued. Second, we are contacting patients multiple and various times throughout the day to schedule their appointments and are even willing to meet with patients after regular business hours if need be.

These challenges highlight the realities of implementing a new intervention, that includes hiring and training staff, in addition to attempting to change practices and behaviors within a complex health care system in a short period of time. We hope that the discussion of our challenges and the strategies that we have implemented thus far to address them will not only inform the field but better equip hospital leaders, administrators and clinical directors who are considering a hospital-based intervention to improve care for patients with SUD.

## Ethics statement

The study protocol, including recruitment, consent, and data collection tools was reviewed and approved by the Institutional Review Board of the University of New Mexico Human Research Review Committee (HRRC 19–174). All participants provide informed written consent prior to enrollment in the study.

## Author contributions

AC: Conceptualization, Data curation, Funding acquisition, Investigation, Methodology, Project administration, Supervision, Writing – original draft, Writing – review & editing. KP: Writing – original draft, Writing – review & editing. JS: Data curation, Investigation, Project administration, Supervision, Visualization, Writing – original draft, Writing – review & editing. TK: Formal Analysis, Writing – original draft, Writing – review & editing. CC: Visualization, Writing – review & editing. VW: Writing – original draft, Writing – review & editing.
